# The Composition and Biochemical Properties of Strophantus (Apocynaceae), with a Focus on *S. sarmentosus*

**DOI:** 10.3390/molecules29122847

**Published:** 2024-06-14

**Authors:** Simone König

**Affiliations:** Core Unit Proteomics, Interdisciplinary Center for Clinical Research, University of Münster, 48149 Münster, Germany; koenigs@uni-muenster.de

**Keywords:** ethnomedicine, cardiac glycoside, bioactivity, steroids, saponins, phenols, alkaloids

## Abstract

The genus Strophantus belongs to the Apocynaceae family of flowering plants which grows primarily in tropical Africa. The plants are widely used in traditional herbal medicine. *S. sarmentosus*, in particular, is used for the treatment of, e.g., joint pain and rheumatoid arthritis, wound infections, head lice, diarrhea, snake bite, and eye conditions. Despite its widespread use, dedicated research characterizing its bioactive plant components is scarce. Investigations have focused mainly on its cardenolides because of their cardioactivity and historical use as cardiotonic. There are also studies concerning the antibacterial, antioxidant, and anti-inflammatory activity of plant extracts. This review summarizes the present knowledge surrounding the biochemical and analytical research on Strophantus, in general, and *S. sarmentosus*, in particular, and describes the current state of the field based on the available scientific literature.

## 1. Introduction

Members of the Apocynaceae family of flowering plants can be found on all continents with a moderate or warm climate. Strophantus grows primarily in tropical Africa, with some species also native in Asia (for the morphology and geographical distribution of all 38 species, see [1]). The genus Strophantus was described by de Candolle in 1802, but Linnaeus had already reported on *S. caudatus* in 1767 [1]. The plants have been used for thousands of years in traditional herbal medicine and, interestingly, also as an arrow poison [2,3]. Their ethnopharmacological significance extends to dermatological diseases and skin care [4]; the treatment of tuberculosis [5] and ulcers [6]; anti-protozoal [7], antibacterial, antioxidant [8], and anti-inflammatory [9] activities; and hypoglycemic effects [10]. 

Medicinal plants contain a variety of substances with potentially useful activities including hydrocarbons, organic acids, polysaccharides, proteins and peptides, fatty acids and essential oils, alkaloids, tannins, saponins, glycosides, bitter substances, and vitamins [11]. Apocynaceae, in particular, are a source of indoline and steroidal alkaloids, cardioactive and cyanogenetic glycosides, saponins, tannins, coumarins, phenolic acids, and triterpenoids (see review [12]; it includes Strophantus, *S. kombe*).

The cardiotoxins which were isolated from Strophantus seeds and which were the main reason for the use of the plant extracts as a poison gained increasing interest in Western medicine as an alternative to digitoxin, the cardiac glycoside from foxgloves, at the turn of the 19th century [13]. They were used in a purified form in clinics to treat congestive heart failure and arrhythmias for many years [2]. Practitioners of alternative medicine still recommend strophantin for its cardiotonic effects [14].

**Figure 1:**
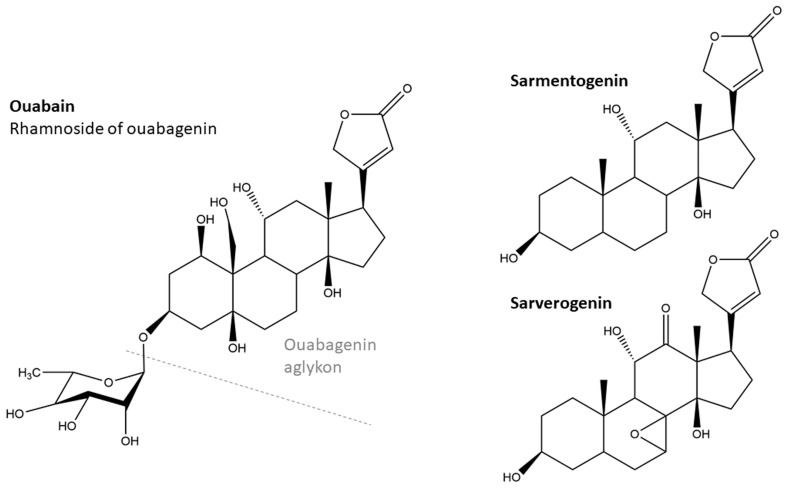
Structure of ouabain, the cardenolide found in *S. gratus* seeds [18], and the sarmentogenin (PubChem CID 6437) and sarverogenin [19] from *S. sarmentosus*.

The interest in Strophantus grew further when, in the late 1940s, a precursor of corticosteroids was needed for the production of cortison and was found in the seeds of *S. sarmentosus* in the form of sarmentogenin (Figure 1) [15]. A difficulty at the time was the distinction of the seeds from different Strophantus species, not all of which contained sufficient amounts of the desired compounds, prompting efforts to classify commercial seeds [16]. For a review of commercially important African medicinal plants, including *S. gratus*, see [17].

In addition to the cardiac glycosides, there are many bioactive compounds in Strophantus, only a few of which have been identified or scientifically characterized. In a collaborative project with a student from Nigeria who was searching for such substances in *S. sarmentosus* (also called spider tresses or poison arrow vine), the state of knowledge was found unsatisfactory, particularly because many publications were not referenced in standard scientific databases like PubMed. *S. sarmentosus* is widely distributed in Nigeria and tropical Africa, in general, and has numerous ethnomedicinal uses including the treatment of joint pain and rheumatoid arthritis, wound infections, head lice, gonorrhea, leprosy, scabies, venereal disease, diarrhea, snake bite, and eye conditions such as conjunctivitis and trachoma [20], but dedicated research studies on this species are scarce. In order to obtain a comprehensive overview, the literature was screened from the viewpoint of an analytical chemist with the goal of generating a summary of the identified molecules in Strophantus extracts and their bioactivity, with a particular focus on the species of interest, *S. sarmentosus*.

## 2. Bioactive Compounds

### 2.1. Cardiac Glycosides and Their Activity

Cardiotoxins such as strophoside, cimarin, and ouabain (Figure 1) have been isolated from *S. kombe*, *S. hispidus,* and *S. gratus*, respectively [3]. Many more of these so-called cardenolides are known; for introductory reading on their structures, biochemistry, and pharmacology, see “Cardiac Glycosides 1785–1985” [21] and review [22] (which mentions *S. kombe*).

Cardenolides consist of steroids and sugars; only steroids containing 23 or 24 carbon atoms have cardiac activity [3]. Figure 1 shows the prominent representative ouabain, which can be isolated from *S. gratus* seeds. Like several members of this substance class, it is toxic when injected into the bloodstream, but not when given orally [3], as a result of its low absorption [23]. Cardenolides can bind to and inhibit Na^+^/K^+^-ATPase, causing cardiotonic activity [24]. For the aglykon of ouabain, ouabagenin (Figure 1), it has also been shown that its oxysterol is a liver X receptor ligand [18].

The glycoside composition in Strophantus seeds has been genetically determined, as proven for four geographically separate chemical forms of *S. sarmentosus* [1,25]. Exemplarily, sarmutoside and musaroside (from aglykon sarmutogenin) were isolated from two rare single plants in Senegal, which differed slightly in composition from typical native Strophantus representatives in that region [26]. Differences in the cardenolide contents of *S. kombe* seeds from Zimbabwe and Malawi were also reported [27].

Analyses of *S. divaricus* proved that cardenolides occur in different parts of the plant and that their concentrations vary; most glycosides were isolated from the leaves in that study [28]. Based on their seed glycosides, Strophantus species were assigned to four groups [1]:

- The ouabain group: *S. gardeniiflorus*, *S. gratus*, *S. thollonii*;
- The sarmentogenin/sarverogenin group: *S. welwitschii*, *S. amboensis*, *S. gerrardii*, *S. congoensis*, *S. petersianus*, *S. courmontii*, *S. sarmentosus*;
- The strophanthidin/strophanthidol/periplogenin group: *S. arnoldianus*, *S. hispidus*, *S. mirabilis*, *S. barteri*, *S. hypoleucos*, *S. mortehanii*, *S. eminii*, *S. kombe*, *S. nicholsonii*, *S. gracilis*, *S. ledienii*, *S. preussii*;
- The divaricoside/caudoside group: *S. caudatus*, *S. divaricatus*, *S. wightianus*.

The dried ripe seeds of *S. kombe* and *S. hispidus* are rich in cardiac glycosides (8–10%) [12] and were an early and important commercial source of these molecules; the purified total mixture of these substances was widely used as an injectable solution (strophantin K) for the treatment of cardiac deficiencies [29,30]. Research has been ongoing for more than 100 years, but new cardenolides are still being isolated, such as glycosides of 17α-strophadogenin in *S. kombe* [29,30]. A dedicated electrospray mass spectrometry (MS) method characterized strophantidin and six different glycosides including cymarin, helveticoside, erysimoside, and neoglucoerysimoside in strophantin K [31].

The strophanthidin glycosides of *S. kombe* seed extracts change upon storage for over 12 months [27]. Cardenolides exhibiting two or three saccharide moieties were degraded presumably by β-glucosidase activities originating from the plant material or lactobacilli, releasing corresponding monoglycosides. They were further degraded into their corresponding aglycons, probably by acid hydrolysis as a result of lactic acid accumulation [27].

In terms of *S. sarmentosus* [32], the Reichstein group conducted many structural investigations in the 1950s and 1960s, identifying 20 glycosides including bipindogenin, lokundjosid, and thollosid in a sarmentoside mixture of water-soluble glycosides (see [19,33] and the references therein). The cardioactivity of sarmentoside extracted from these seeds was tested on rabbit heart preparations [34]; it compared favorably to digoxin, increasing the force and rate of heart contractions. These effects were antagonized by a potassium chloride solution.

The cardioactivity of the bark extract of *S. cumingii* and its sub-fractions was determined on isolated frog hearts [35]. The hexane fraction was the most cardioactive, with a maximum of a 31% increase in the force of contraction and 38% increase in the frequency of contraction. The non-polar fraction of the crude extract from the bark elicited a positive inotropic and negative chronotropic effect on the hearts.

*S. hispidus* is used in the treatment of myocardial infarction in Nigerian ethnomedicine. A study of the hearts from male Wistar rats pretreated daily with *S. hispidus* extract for 14 days before an isoprenaline hydrochloride injection (ISO) demonstrated that the pretreatment not only protected against an excessive release of cytochrome c but also resulted in decreased caspase 3 activation, which prompted a decrease in excessive apoptosis [36]. The reduction in lipid peroxidation levels in the ISO-induced myocardial infarction in the rats correlated with the decrease in their creatine kinase and aspartate aminotransferase levels. 

In another study, the use of *S. hispidus* against ischemia-reperfusion myocardial infarction and renal artery-occluded hypertension in rats suggested that the ethanolic extract had significant cardiac protective and anti-hypertensive activity [37]. Infarction size, blood pressure, and heart rate were reduced.

Modern analytical technologies such as high-resolution MS and nuclear magnetic resonance spectroscopy have accelerated the identification process of natural substances in recent years and led, e.g., to the description of six new cytotoxic cardenolide glycosides from *S. boivinii* (boivinides), which, on a side note, exhibited antiproliferative activity in a human ovarian cancer cell line [38]. In fact, several cardiac glycosides were found to exert potent antitumor activity; ouabain, e.g., showed antiproliferative effects on SW13, H295R, and five primary adrenocortical tumor cells [39]. Of 109 isolated and identified Apocynaceae cardenolides, about a quarter had the capability to regulate cancer cell survival (for a review with limited information about Strophantus, see [40]). *S. gratus* and *S. caudatus* extracts, e.g., showed no anticancer and antifungal activity in a study of 23 Indonesian plant families [41]. The choice of the sugar moieties in position C-3 is fundamental for their large growth inhibition against cancer cells, and their cytotoxic effects are decreased with the length of their sugar chain [40]. 

Divaricoside from *S. divaricatus* inhibited cell growth in a dose- and time-dependent manner in SCC2095 and oral squamous cell carcinoma OECM-1 OSCC cells [42]. It induced autophagy, S and G2/M phase arrest accompanied by a downregulation of phosphorylated CDC25C, CDC25C, and CDC2 in SCC2095 cells, and apoptosis by activating caspase 3 and downregulating the expression of Mcl-1. These findings suggest its translational potential as a therapeutic agent for OSCC treatment [42].

Testing the hypothesis that they may provide patients with baseline protection against cancers and/or be an adjuvant treatment for chemotherapy-resistant cancers, 27 popular herbal infusions widely used in Nigeria for diabetes were studied on a panel of liver (HepG2), colon (Caco2), and skin (B16-F10) cancer cells [43]. The results showed that an *S. hispidus* stem extract was preferentially toxic against the human colon carcinoma Caco2 cell line. It was concluded that its regular intake by diabetic patients may provide baseline protection against colon cancer.

Not only do cardenolides exhibit bioactivity, but so do steroid core structures. For example, cytotoxic steroids from the twigs, stem, and leaves of *S. divaricus* were described [44,45].

Interestingly, on a side note, Apocynaceae cardenolides have been detected in monarch butterflies [46]. These insects sequester cardiac glycosides from milkweed plants as part of their adaptive strategy. These substances elicit vomiting in birds, who learn to avoid this prey.

### 2.2. Triterpene Glycosides and Other Substances

In contrast to cardiac glycosides, triterpene glycosides have not been as extensively investigated, although Strophantus extracts have long been used as emetics, and for the treatment of respiratory diseases, by native Africans [2]. Saponins (water-soluble foam-forming plant components) are credited with emetic, secretolytic, and expectorant activity. For a recent review on Strophantus saponins and the triterpene glycosides in *S. gratus*, see [2].

**Figure 2:**
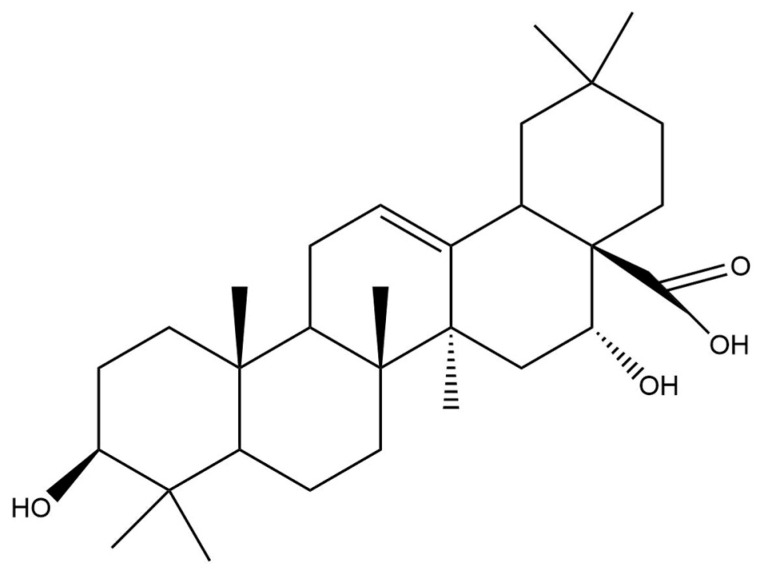
Structure of echinocystic acid, which was isolated from fermented seed extract of several Strophantus species including *S. sarmentosus* [2].

In *S. sarmentosus* and other Strophantus species, echinocystic acid (Figure 2) was isolated from fermented seed extract as a core structure, e.g., of the bidesmoside in *S. gratus* [2]. Echinocystic acid, as a natural extract, is widely used in the treatment of inflammatory diseases and reported to alleviate ischemia/reperfusion injury via inhibiting the JNK signaling pathway in mice [47]. 

Phytochemical screening for the various substance groups in Strophantus plant extracts is typically performed using specific assays [8,10]. Different plant parts vary in their composition; a *S. sarmentosus* stem methanol extract, for instance, tested positive for flavomoids, saponins, phenolics, tannins, carbohydrates, glycosides, alkaloids, steroids, and terpenoids, while a leaf extract only showed five of these substance classes [48,49]. 

The gas chromatography (GC)-MS of the seed oils of *S. kombe* characterized their components as mainly fatty acids, especially oleic acid and linoleic acid, and phytosterols, with the latter representing intermediates of cardenolide biosynthesis [30]. In the seed oil of *S. sarmentosus,* the major component acids were palmitic (12%), oleic (38%), and linoleic (30%) acids; its minor components included stearic acid (9%), some saturated acids (4%) higher than stearic, and an unsaturated hydroxy-acid (7%) not previously reported [50]. In addition, triglycerides and 2-monoglycerides have been investigated in that species [51]. Another GC-MS study of methanol extracts suggested the presence of six more compounds, such as octadecyl vinyl ether and hexadecanal diisopentyl acetal, without validation [49]. The compound 2-hydroxy-4-methoxy-benzaldehyde has been described in the methanolic extract of *S. wallichii* [52].

**Figure 3:**
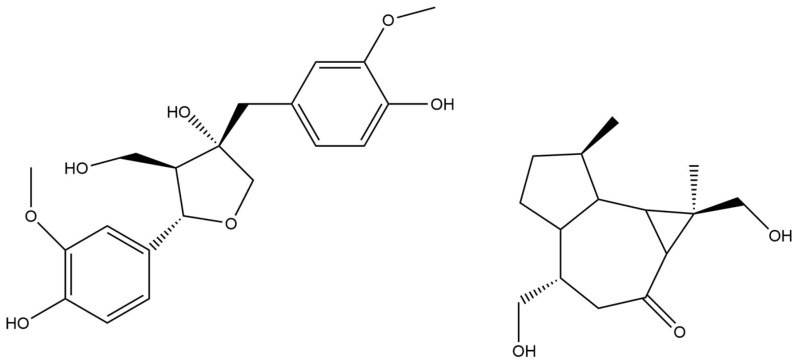
Structures of olivil (left) from *S. gratus* [53] and strophantoid A from *S. divaricatus* [56].

Lignans from *S. gratus* stem bark (pinoresinol, olivil; Figure 3) [53] and cyclitols from its leaves (bornesitol and dambonitol, generally found in Apocynaceae) [54,55] have been structurally analyzed. From the stem and roots of *S. divaricatus,* sesquiterpenoids (Figure 3) were characterized, one of which (neridienone A) exhibited significant cytotoxicity against human cancer cell lines [56,57]. 

The seeds of *S. kombe* and *S. hispidus* contain, besides cardioglycosides, about 30% oil and other constituents such as kombic acid, alkaloids, choline and trigonelline, resin, mucilage, and calcium oxalate [12]. In *S. hispidus,* the chromatographic finger-printing of a methanol stem bark extract detected seven major compounds including ascorbic acid, quercetin, resorcinol, and gallic acid, which were present in large amounts [58,59]. A *S. hispidus* stem ethanolic extract contained polyphenol, flavonoids, tannins, alkaloids, terpenoids, and saponines [60]. In a study of plants used against diabetes mellitus [61], *S. hispidus* exhibited much smaller quantities, compared to other plants, of several phenolic compounds such as rutin, gallic acid, and ellagic acid.

## 3. Bioactivity

### 3.1. Toxicity and Mutagenicity

Despite the fact that numerous plants are used in ethnomedicinal practice, scientific studies about their undesirable and toxic effects are limited. A report concerning plants used in Guinean traditional medicine [62] mentioned the adverse or toxic effects of *S. hispidus* and associated them with the presence of cardiotonic heterosides, which are known to have a narrow therapeutic margin.

A sub-chronic toxicological investigation of an aqueous root extract of *S. hispidus* in rats showed no significant alterations in the body weight of treated compared to control rats [63]. At doses of 500 and 1000 mg/kg, the treated rats experienced a significant increase in white blood cells and decrease in liver weight, but the extract otherwise demonstrated a good safety profile for oral administration. 

A study of the sub-chronic effects of aqueous and ethanol extracts of *S. hispidus* in normal rats indicated that higher doses were dangerous to the liver and heart, with the ethanol extract posing a greater risk [64]. The plant extracts caused significant increases in liver proteins (alanine aminotransferase, aspartate aminotransferase, alkaline phosphatase, γ-glutamyltransferase, albumin) and heart enzymes (lactate dehydrogenase, creatine kinase), whereas liver markers decreased significantly in relation to the control. These alterations were, in most cases, seen at doses higher than 800 mg/kg. Histology revealed mild to marked morphological changes with increasing dosages.

Over half of the most frequently used medicinal plants (110 of 53 families) in Ghana showed moderate to high mutagenicity, but the Strophantus representative in this project, *S. gratus*, was not among them [65]. 

It is important to mention that the high lipophilicity of cardiac glycosides supports their harmful effects on the human body; the inhibitory potency of cardiotonic steroids on myocardial Na^+^/K^+^-ATPase increases with their increasing lipophilicity [66].

### 3.2. Anti-Inflammatory, Antibacterial, and Antioxidant Activity

Free radicals and oxidative stress play a major role in the development of tissue damage and pathological processes which result in inflammation. *S. gratus* is used in Ghana for managing inflammation-related conditions. A study detected (a carrageenan-induced paw edema model in 7-day old chicks) anti-inflammatory and antioxidant activities (via a phosphomolybdenum assay) in the sub-ethyl acetate fraction of a leaf ethanolic extract [67]. 

The leaves and root of *S. preusii* proved to be potent natural antioxidants, which justified their traditional use in the management of stress-related diseases. The antioxidant and acetylcholinestrase (AchE)-inhibitory properties of methanol extracts (leaves, stem, root) were evaluated by standard in vitro methods, viz., 2,2-diphenyl-1-picrylhydrazine (DPPH), nitric oxide (NO), hydroxyl radical (OH-), and hydrogen peroxide (H_2_O_2_) radical scavenging assays, as well as though reducing power, Fe^2+^/ascorbate-induced lipid peroxidation (LPO), and AChE inhibition assays with catechin as the standard [68]. High phenolic and flavonoid contents were found in the aerial parts of this plant. All extracts showed antioxidant activities in vitro. The leaf extract had the highest DPPH, H_2_O_2_, and OH radical scavenging ability and the root extract the most reducing power. All extracts significantly inhibited LPO in rat livers by 30–40% and in rat brains by 30–70%, similar to the standard. Only the stem extract produced significant NO scavenging effects. The percentage inhibition of AChE activity was significant for the leaf and root extracts [68].

Several studies on *S. hispidus* reported that the entire plant (stem bark, leaves, roots) had therapeutic applications including the treatment of skin diseases, gonorrhea, dysentery, leprosy, diabetes, edema, malaria, ulcers, rheumatism, and urine retention [6]. Significant antioxidant activity, using DPPH, was detected for a methanol extract of *S. hispidus* stem bark [59]. Its in vitro antibacterial activity was better against Gram-negative bacteria (*Escherichia coli*, *Pseudomonas aeruginosa*) than *Staphylococcus aureus* in that work. Also, in another study, aqueous and organic (cyclohexane, chloroform, ethyl acetate, ethanol) stem extracts of *S. hispidus* exhibited strong and broad-spectrum antimicrobial activity against these three microbes [60]. Only the ethanolic extract had appreciable antioxidant activity, as evaluated using DPPH. In contrast, another study did not find any activity against ten Gram-positive and three Gram-negative (*Proteus vulgaris, Klebsiella pneumoniea*, *E. coli*) bacteria [8], and the low antioxidant activity of the extract was measured in comparison to a reference antioxidant (ascorbic acid).

A total of 211 plant species from 56 families are used in West Africa for several skin conditions such as aphthous ulcers, burns, eczema, scabies, sores, and wounds (for a review, see [4]). Among these, *S. hispidus* leaf, stem bark, and root methanol extracts showed significant wound-healing effects, as well as antimicrobial (against two Gram-positive, two Gram-negative bacteria, and a fungus) and antioxidant (DPPH) properties [69]. Wound tissues treated with the extracts exhibited improved collagenation, re-epithelization, and rapid granulation formation compared to untreated wound tissues.

Cytoprotective properties are typically associated with the release of free radical scavengers. Anti-ulcer activity was shown by a root ethanolic extract investigated in albino rats sporting three ulcer models (ethanol, HCl, and pylorus ligation) [6]. The results from the ethanol-induced ulcer model indicated a marked reduction in ulceration compared to misoprostol, a standard drug. The results from the HCl-induced ulcer model showed less ulceration than cimetidine. Moreover, in the pyloric ligation-induced ulcer model, the *S. hispidus* extract demonstrated an effective reduction in gastric ulcers compared to omeprazole. The reduced ligation of gastric acid and its accumulation in the stomach of the albino rats were also observed. Investigations conducted in the aspirin-induced ulcer model using an aqueous antiulcer drug formulated with *S. hispidus* as a constituent found that, in albino rats, the formulated drug markedly reduced ulceration compared to omeprazole [6].

Extracts from multiple plants are often combined in ethnomedicine to achieve synergistic effects. As aqueous extracts from *S. hispidus* (roots) and *Aframomum meleguta* (seeds) are topically co-administered to the nasal cavities for the management of chronic sinusitis, a study assessed the anti-inflammatory, antimicrobial, and antioxidant effects of these preparations [9]. The individual plant extracts showed comparable potency to that of the mixture with regard to their antimicrobial activity and DPPH radical scavenging activity. The anti-inflammatory activity (inhibition of carrageenan-induced 7-day old chick feet edema) evoked by the mixed extracts was, however, greater than the sum of the individual potencies of the two extracts [9].

Only two studies, both from Nigeria, have investigated *S. sarmentosus* in more detail with regard to the anti-inflammatory, antimicrobial, and cytotoxic activities of its different plant extracts [48,49]. Crude extracts of its leaf, root bark, and stem (n-hexane, dichloromethane, methanol) had a significant anti-inflammatory effect against the egg albumin-induced inflammation of rat paw edema in comparison to a standard drug (aspirin), with root bark working best [49]. The highest dose of 400 mg/kg was lethal to the animals. 

In another study [48], cold extracts (hexane, ethyl acetate, methanol) of plant parts (leaf, stem, roots) demonstrated considerable activity against both Gram-positive and Gram-negative bacteria, as well as fungi. Thereby, *Candida albicans, Staphylococcus typhimonium, S. aureus,* and *P. aeruginosa* were inhibited the most, while the least susceptible were *K. pneumonia* and *Bacillus subtilis*. The stem methanol and the root ethyl acetate extracts were the most active and comparable to a standard drug (gentamycin). The cytotoxic activity (brine shrimp method) of the extracts ranged within a medium toxic level according to Clarkson’s toxicity index.

Part of the antioxidant capacity of a plant may originate from its endosymbiotic species. In a study of 292 morphologically distinct endophytic fungi isolated from 29 traditional Chinese medicinal plants, including *S. divaricus*, the antioxidant capacities of the endophytic fungal cultures were significantly correlated with their total phenolic contents, and phenolics were major antioxidant constituents of the endophytes [70]. 

### 3.3. Hypoglycemic Effects

In type 2 diabetes mellitus, the inhibition of α-glucosidase is a useful treatment to delay the absorption of glucose after meals. In a study, the α-glucosidase inhibitory activity of 80% ethanol extracts of the leaves and twigs of some plants from the Apocynaceae, Clusiaceae, Euphorbiaceae, and Rubiaceae families were determined. Compared with the control acarbose, 37 samples out of 45 were shown to be more potent α-glucosidase inhibitors, including the Strophantus representatives (*S. gratus* folium, *S. caudatus* cortex) [71].

For *S. hispidus*, contrasting results for and against its hypoglycemic activity have been published. One investigation demonstrated the hypoglycemic and antioxidant effects of its aqueous root extract in streptozotocin-induced diabetic rats using the activities of its superoxide dismutase, total peroxidases, γ-glutamyl transferase, glutathione-S-transferase, glutathione peroxidase, and glutathione reductase, as well as the concentrations of its glucose, glutathione, vitamin C, nitric oxide, total thiols, and malondialdehyde, as indices [72]. The concentrations of blood glucose, NO, and malondialdehyde were significantly decreased in all treatment groups that received the extract. These results were supported by other work proving the beneficial antidiabetic activity of this species’ aqueous root extract in rats [73]; the extract produced a day-dependent reduction in glucose level. It significantly increased the levels of high-density lipoprotein, total protein, catalase, superoxide dismutase, and reduced glutathione in rats, and reduced their levels of triglycerides, low-density lipoprotein, total cholesterol, aspartate transaminase, alanine transaminase, alkaline phosphatase, bilirubin, creatinine, and urea compared to diabetic control rats. It significantly inhibited α-amylase and α-glucosidase enzymes compared to acarbose. Another study found significant concentration-dependent hypoglycemic effects of both the ethanol and chloroform extracts of *S. hispidus* leaf, stem, and root in Wistar male rats in streptozotocin-induced diabetes mellitus, with the ethanol leaf extract working best [10]. Chloroform root extracts exhibited more prolonged hypoglycemic effects. 

Furthermore, aqueous and ethanol extracts of *S. hispidus* stem bark showed hypoglycemic, anti-hyperlipidemic, and antidiabetic activities [74]. There was a significant progressive decrease in fasting blood glucose concentration in the 2nd–12th weeks and 4th-12th weeks in normal and diabetic treated rats, respectively. Total cholesterol, low-density lipoprotein, and triglyceride levels were lowered significantly by these extracts in normal and diabetic treated rats, whereas high-density lipoprotein levels were elevated in diabetic treated rats. The activity of serum α-amylase, the inhibition of which is an important therapeutic target in the regulation of postprandial increases in blood glucose in diabetic patients, was also elevated by the extract in diabetic treated rats in that work. 

In contrast, in a study of the α-amylase inhibitory and antioxidant potential of selected herbal drugs used for the treatment of diabetes by traditional healers in Nigeria, *S. hispidus* methanol crude extracts did not show potent free radical scavenging or efficient reducing power compared to other plant samples and exhibited no α-amylase inhibition [75]. In another investigation of aqueous extracts of 27 Nigerian plants for their in vitro effects on the glutathione levels within HepG2 cells, P-gp-mediated Rh-123 efflux activity in Caco-2 vincristine-resistant cells, and the modulation of glibenclamide transport in Caco-2 monolayers [61], only three plants had a significant effect at the same level as the reference drug verapamil, and the Strophantus representative *S. hispidus* was not among them. 

### 3.4. Anti-Nociceptive Effects

Decoctions of the root of *S. hispidus* are highly valued in African herbal medicine. A study evaluated the anti-nociceptive effect of its aqueous root extract in Swiss albino mice using various models (acetic acid-induced writhing, formalin, Haffner’s tail clip, hot plate, tail immersion tests) [76]. The extract was administered orally to the animals and possessed, in each of the models, a significant anti-nociceptive effect in a dose-dependent manner. The involvement of opioid and dopamine receptors in anti-nociception was established. The effect of the extract was comparable to that produced by peripheral analgesics, NSAIDs (aspirin), and centrally acting analgesic opioids (morphine), which were used as positive controls. These findings showed that *S. hispidus* provides anti-nociceptive effects mediated both peripherally and centrally.

Another work reported the anti-nociceptive, anti-inflammatory, and anti-ulcerogenic properties of an ethanol root extract of *S. hispidus* [77]. Its anti-nociceptive activity was evaluated using acetic acid-induced writhing and formalin tests in mice. Carrageenan- and egg albumin-induced rat paw edema tests were used to investigate its anti-inflammatory actions, while its antiulcer activity was investigated using ethanol-, HCl-, and pyloric ligation-induced gastric ulcer models in rats. The extract, given orally, produced a significant inhibition of the writhing reflex and attenuated the formalin-induced early and late phases of nociception. It caused a significant inhibition of edema development in the carrageenan and egg albumin models and showed potent antiulcer activity [77].

### 3.5. Anti-Venomous Activity

Snakebite envenomation causes about 5–10,000 deaths and results in more than 5–15,000 amputations in sub-Saharan Africa alone every year. Antiserum is not easily accessible in these regions; thus, more than 80% of all patients seek the help of ethnomedicine. It is important to elucidate whether medical plants contain compounds that act against the necrosis-inducing enzymes of snake venom. A total of 226 extracts from 94 traditionally used plant species from Congo, Mali, and South Africa were tested in hyaluronidase, phospholipase A2, and protease enzyme bioassays using *Bitis arietans* and *Naja nigricollis* venoms as enzyme sources [78]. Forty plant species showed more than a 90% inhibition in one or more assay, but the Strophantus representatives (*S. sarmentosus*, *S. speciosus*) had low to medium effects in comparison [78].

In another work, aqueous extracts of the leaves of *S. gratus* and *S. hispidus* prolonged the time taken to clot for blood treated with the venom of *Echis carinatus,* with *S. hispidus* being the most potent [79].

### 3.6. Anti-Phytoviral, Anti-Herpetic, Anti-Trypanasomal, Anti-Protozoal, Anti-Malarial, and Hydroxynitrile Lyase Activities

The tuber necrotic strain of potato virus Y (PVYNTN) causes widespread disease and has severe negative effects on the growth and yield of plants. Ethanolic extracts of the fruits and leaves of *S. speciosus* showed a significant inhibition of PVYNTN in vivo and in vitro and, thus, have the potential to be used as an anti-phytoviral treatment [80].

Aqueous ethanol (80%) extracts of six plants used traditionally for the treatment of malaria, including *S. eminii,* were screened for their anti-malarial activity [81]. This extract exhibited low activity in mice inoculated with red blood cells parasitized with *Plasmodium berghei*. It was innocuous to the mice and not toxic up to 2400 mg/kg body weight, suggesting it may be safe for short-term use.

An investigation reported the significant anti-trypanosomal activity of an *S. sarmentosus* methanolic stem extract with a median lethal dose of 100 mg/kg body weight in mice, but it did not inhibit *P. vulgaris, E. coli, S. aureus,* and *Enterobacter* spp. [82]. 

In an ethnobotanical survey of 41 Guinean plant species widely used in the traditional treatment of fever and malaria, *S. hispidus* showed no in vitro anti-protozoal activity or cytotoxicity on MRC-5 cells [7].

Herpes simplex virus infection is associated with oral mucocutaneous lesions and/or genital infections. A methanolic extract of *S. hispidus* (stem bark) had good anti-herpetic activity against anti-herpes simplex virus (1 and 2), but it was also highly toxic [58].

Hydroxynitrile lyases are used for the synthesis of enantiomerically pure cyanohydrins, which are of technical importance as building blocks in the pharmaceutical and fine chemical industries. In over 3000 plant species, cyanohydrin is broken down by this enzyme, but no such activity or cyanogenic properties were found in *S. amboensis* and other members of the Apocynaceae [83].

## 4. Methods

Information was obtained by searching the literature using the SciFinder and Google Scholar tools, as well as regular internet browsers, for publications with the search term Strophantus on 30 April 2024 and in early May. Since the resulting matches were few in number, a general overview of all publications was obtained and refined based on recent reviews and publications. Articles in English and German were read, the very few published in other languages were evaluated using their abstracts. *S. sarmentosus* is printed in bold throughout the manuscript for the faster locating of the species of interest. 

## 5. Conclusions

Strophantus is a plant of high ethnomedicinal importance and considerable commercial interest. Its cardiotoxin components have been the focus of many past investigations. Scientific research regarding further isolated bioactive compounds from this genus is, however, sparse and, in some cases, contradictory. Different extract qualities may be associated with the varying compositions of uncultured plants collected in the wild. A number of studies report the biochemical properties of plant extracts, but the path to obtaining an individual purified substance is long and not often taken. Many species of the genus are under-researched, including *S. sarmentosus*. Nevertheless, properties such as its antibacterial, antioxidant, and even anti-cancerous and anti-venomous activity have been reported for aqueous and organic extracts of its plant parts, and it is promising to study them further.
